# Positive Reinforcement-Based Training for Self-Loading of Meat Horses Reduces Loading Time and Stress-Related Behavior

**DOI:** 10.3389/fvets.2019.00350

**Published:** 2019-10-10

**Authors:** Francesca Dai, Alessandro Dalla Costa, Lebana Bonfanti, Claudia Caucci, Guido Di Martino, Roberta Lucarelli, Barbara Padalino, Michela Minero

**Affiliations:** ^1^Dipartimento di Medicina Veterinaria, Università degli Studi di Milano, Milan, Italy; ^2^Istituto Zooprofilattico Sperimentale delle Venezie, Legnaro, Italy; ^3^Department of Agricultural and Food Sciences (DISTAL), University of Bologna, Bologna, Italy

**Keywords:** horse, transport, welfare, training, behavior

## Abstract

The present work aimed to evaluate the efficacy of a self-loading training using positive reinforcement on stress-related behaviors shown by meat horses during loading procedures into a truck. Thirty-two meat horses (*M* = 18; *F* = 14; 6 month-old) were included in the study. All horses had limited interactions with the farmer and were not used to be restrained nor lead by halter. Horses were divided in two groups: Control Group (C; *N* = 14) and Training Group (T; *N* = 18). T horses were trained to self-load: in order to teach the horses to enter into the truck, a targeting training technique throughout a shaping process was applied. Training sessions were performed three times a week, from 9:30 a.m. to 1:30 p.m. and from 2:30 p.m. to 4:30 p.m., for 6 weeks; training was then repeated once a week to maintain the memory until the transport toward a slaughterhouse. The loading phase was video-recorded and loading time was directly recorded using a stopwatch. All horses were transported to the same slaughterhouse in 14 different days using the same truck. Behavior was subsequently analyzed with a focal animal continuous recording method. Loading time was shorter in T horses (mean ± SD = 44.44 ± 47.58 s) than in C horses (mean ± SD = 463.09 ± 918.19 s) (*T*-test; *p* = 0.019). T horses showed more forward locomotion toward the truck than C horses (*T*-test; *p* = 0.029). Our preliminary findings suggest that self-loading training may be useful to mitigate loading-related stress in meat horses, minimally socialized with humans.

## Introduction

Loading is considered one of the most stressful stages of animal transport (1, 2), involving new experiences such as being handled by humans, being mixed with unfamiliar animals and entering a novel environment (the vehicle) (3). Several studies highlighted the relationship between loading and transport-related problems in sport horses: many horses exhibit strong reactions during loading, which can lead to injuries to the handlers (including rope burns, lost fingers, broken bones, or bruises, and bleeding) and to the animal (including lacerations to the head from banging into the trailer, scrapes and cuts on the legs, broken legs from falling, or even a broken back if the animal falls backwards) (4). Horses subjected to transport stress can be more susceptible to a number of disorders, such as pneumonia, diarrhea, colic, laminitis, injuries, and rhabdomyolysis (5), which, not only severely affect their welfare, but can also be costly for the owner. Whilst only few studies concentrated on the incidence of transport related stress on equine meat quality (6, 7), a large body of research describes how transport stress negatively affects meat quality in several other species [see for review (8, 9)]. Not only loading problems are a source of stress for the animals, but also costly in time for the personnel involved in the loading, endangering the economic benefit for the owner (10, 11). Preparation of animals to transport through the adoption of appropriate management measures plays an important role in mitigating transport-related stress (3). Reducing pre-transport stress decreases the probability of compromising animal welfare during the transport phase (3). Among suggested pre-transport measures reported in the literature, we can find: adequate route planning (12), proper evaluation of animal-related factors, such as species, breed, age, temperament, behavior, and health status (3), appropriate handling during loading and unloading (i.e., collection of animals, weighing, loading, penning should be done in calm and gentle manner to minimize stress) (3). Rearing conditions and previous experiences, both with handling and with transport procedures, have a high impact on the stress response of animals during handling at loading (13–15). In sport horses, studies suggest that habituation to loading and traveling significantly reduces the likelihood that horses develop transport related behavioral problems and injury (16, 17). Loading training using positive reinforcement [consisting of reward delivery in response to the desired behavior (18)] also seems to reduce loading time and stress during loading (14, 19). Finally, it has been proven that self-loading techniques reduce the likelihood of horses showing behavioral problems (such as attempting to escape, rearing, kicking, pulling back, standing still, pawing) at loading (16, 17). While there is a body of literature reporting about the effect of training to load in sport horses [i.e., (4, 11, 14, 16)], nothing has been published on meat horses completely naïve to transport. This study population deserves great attention because horses kept for meat production are generally transported to the slaughterhouse without any training [see (20–22) for a review], with adverse effects on their welfare (7, 23, 24). It was consequently hypothesized that self-loading training would reduce the time to load, stress-related behavior, and behavioral problems during loading in meat horses. The present work aimed to evaluate the efficacy of a self-loading training technique on stress-related behaviors and loading problems in meat horses loading into a truck.

## Materials and Methods

### Farm and Animals

The study was conducted at a meat horse farm located in North Eastern Italy. Thirty-two Spanish Breton meat horses of both sexes (*M* = 18; *F* = 14), aged 15 ± 2.79 months (min = 12 month; max = 24 months), were randomly selected to be included in the study. Horses were originally imported from Spain at 6 months of age and remained at the farm for fattening until the age of 12–24 months. At the beginning of the experimental procedures, horses had been on farm for 4 months. Upon arrival to farm, animals were randomly divided by the farmer into pens balanced for gender. Two pens were randomly selected by the researchers to enter the study (pen 1: 14 horses; pen 2: 18 horses; density = 2 m^2^ per horse). Groups were kept stable throughout the fattening period and the experiment. The stable had deep litter bedding and, when climatic conditions were favorable, the horses had access to an outdoor area with concrete floor (128 m^2^ per group). The outdoor area was connected to a load lane (14.5 m long), leading to a concrete ramp (6 m long, 8% of slope) and the trailer (trailer ramp: 1.5 m long, 10% of slope). Horses had *ad libitum* access to total mixed ration and all pens were equipped with automatic drinkers. All horses had limited interactions with the farmer and were not used to be restrained, lead by halter or head collar nor transported. Horse interactions with the farmer included daily check of the animals from outside the pens and feeding using a truck. No physical interaction normally occurred.

### Training Protocol

Animals in pen 1 were categorized as Control Group (C; *N* = 14) and animals in pen 2 were categorized as Training Group (T; *N* = 18). Horses in the Training Group were subjected to a non-aversive training to self-load. A target training using operant conditioning [a learning method occurring through rewards and punishments for behavior (25)] and positive reinforcement (a nibble of flaked corn) was applied. Target training (4) consisted of training the horses to follow the target (a yellow stick) that was progressively moved toward the truck (IVECO EUROCARGO 75E17). Training was subdivided into two phases; the first inside the stable and the second in the outdoor area. Horses were firstly habituated to receive food from the trainer's hand, then they were reinforced when touching the target placed in front of their nose. After the horses touched the target every time for five repetitions in a row, the target was moved 50 cm in front and slightly laterally to their nose; horses were reinforced when touching the target after having followed its movement. When all the horses in the pen were able to follow the target for five steps inside the stable, they were allowed to access the outdoor area. In this second phase, a shaping technique [the differential reinforcement of successive approximations toward a target behavior (25)] was applied to train the horses to load into the truck. Horses were firstly reinforced when following the target in the load lane connecting the outdoor area to the truck; then they were reinforced only when following the target up to the start of the vehicle loading ramp. Finally, the truck was positioned at the end of the ramp with opened doors and handfuls of flaked corn on the floor. Horses were led to the ramp with the help of the target and were then left free to load; three horses at a time were trained together in order to take advantage of their gregarious behavior. When loading on the truck, horses were left free to explore it, eat the food located inside the truck and unload to return back to the outdoor pen with the other horses. The training was considered successful when the horse entered the trailer each time it was allowed to do so for 1 week (i.e., three times). Each of the two phases lasted 3 weeks; training sessions were performed three times a week, from 9:30 a.m. to 1:30 p.m. and from 2:30 p.m. to 4:30 p.m.; training was then repeated once a week to maintain the memory until the transport to the slaughterhouse. All training procedures were performed by the same experimenter (FD).

### Loading to Transport

Horses were transported to the slaughterhouse according to the farm's ordinary routine. Two to four horses at a time were transported with the same truck used during the training phase. Transport procedures took place in the afternoon (~4 pm) on different days from April to October 2018, for a total of 14 transports. The farm manager conducted all the transport phases (loading, travel, and unloading) following the usual farm procedures. Usual loading procedures adopted on the farm involved minimal handling of small groups of horses, in order to exploit their gregarious behavior: moving fences to let horses enter the loading lane, inciting horses from behind using voice and moving a stick only when they refused to move.

### Behavioral Evaluation

For each horse, the loading phase of the transport to the slaughterhouse was video-recorded using a digital video camera (Canon Legria HFR88), controlled by the experimenter. The ethogram used for behavior analysis was adapted from Yngvesson et al. (10) (Table 1). Horse behavior was analyzed with a focal animal continuous recording method, using the software Solomon Coder beta 17.03.22. Duration of different behaviors was recorded. The latency to load (from the beginning of the procedures until the horse had all four feet on the trailer) was directly recorded using a stopwatch.

**Table 1:** Ethogram for the evaluation of horse behavior during loading [modified from (10)].

**Behavior**	**Description**
Forward walk^*^	The horse walks toward the trailer
Forward trot^*^	The horse trots toward the trailer
Forward gallop^*^	The horse gallops toward the trailer
Backwards^§^	The horse moves away from the trailer
Standing^§^	The horse stands on the four legs
Turn back^§^	The horse tries to turn all its body in the opposite direction of the trailer
Still^§^	The horse stops moving, digging in its heels, refusing to proceed
Rear^§^	The horse rears with its front legs
Kick^§^	The horse kicks, one or two legs is lifted and moved rapidly and forcefully
Mount^§^	The horse mounts the horse in front of him/her
Paw^§^	The horse rises a foreleg and scrapes the floor
Sniffing^§^	The horse sniffs the ground
Defecate^§^	The horse drops manure
Urinate^§^	The horse drops urines
Other	Any other behavior

* *High frequencies of forward locomotion behaviors were considered to be associated to low stress*.

§ *High frequencies of these behaviors were considered to be stress-related*.

### Statistical Analysis

Behavioral data were analyzed with SPSS Statistic version 25 (IBM Corp.). Walk and Trot were considered as Forward Locomotion. Based on the total length of the observation of the video recordings, durations of behaviors were calculated as percentage of total observation time (proportional duration time). Descriptive statistics [median, mean, and standard deviation (SD) of proportional durations] was performed. Data were tested for normality and homogeneity of variance using Kolmogorov–Smirnov and Levene test, respectively. Behavioral data were not normally distributed; therefore, a log transformation was applied to approximately conform them to normality. A two-tailed *t*-test was applied to identify differences in duration of different behaviors between Control Group and Training Group. Differences between groups in latency to load were analyzed with a two-tailed *t*-test. As basic assumptions for the *t*-test (equal standard deviations) were not met for the comparison of latency to load between control horses showing stress-related behaviors with other horses of the control group, a Mann–Whitney test was used. Differences were considered to be statistically significant if *p* < 0.05.

## Results

No horses showed intense fearful reactions toward the trainer, the target nor the trailer. All horses in the training group reached the success criterion (i.e., entering the trailer each time they were allowed to do so for 1 week) at the end of the sixth week of training.

**Figure 1:**
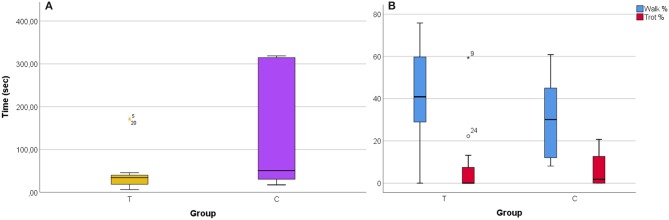
Boxplots reporting data distribution of: **(A)** time needed to load in Training Group (T) and Control Group (C); **(B)** Percentage of time spent walking and trotting in Training Group (T) and Control Group (C). The band inside the box represent the median; the whiskers represents the lowest datum still within 1.5 interquartile range of the lower quartile, and the highest datum still within 1.5 interquartile range of the upper quartile; mild outliers are presented as °, while extreme outliers as *.

Time needed to load was significantly different between the two groups (*T*-test; *t* = −2.472; *p* = 0.019); trained horses needed significantly less time to load (min 6.4 s, max 172.8 s, median 34.3 s, mean 44.4 ± 47.6 s) than control horses (min 17.4 s, max 262.3 s, median 50.7 s, mean 463.1 ± 918.2 s) (Figure 1A).

Horses in the Training group showed more forward locomotion toward the truck (walk and trot) than control horses (*T*-test; *t* = 2.299; *p* = 0.029) (Figure 1B). Duration of other behaviors did not differ between the two groups. However, it is worth noticing that some stress-related behaviors were manifested nearly exclusively in control group: rear (one horse in the control group, for 0.06% of the time), kick (one horse in the control group, for 0.02% of the time), mount (one horse in the control group, for 1.02% of the time), paw (four horses in the control group, for 1.39 ± 0.92% of the time, and one horse in the training group, for 7.64% of the time), defecate (three horses in the control group, for 1.71 ± 0.49% of the time). Mean loading time for horses showing stress-related behaviors was significantly higher than for the other subjects of the control group (1234.08 ± 1258.44 vs. 34.76 ± 15.83 s; Mann–Whitney Test *p* = 0.012).

## Discussion

This study evaluated the efficacy of a self-loading training using positive reinforcement on behavior and loading duration in meat horses. The results supported partially our hypothesis. Self-loading training significantly reduced the time to load and increased the percentage of forward movement (walk/trot), but we did not find a difference between the frequencies of the stress related behavior. The latter finding may be due to the fact that the horses were not completely naïve to transport, since they experienced the travel from Spain to Italy, and consequently had already been exposed to at least one loading procedure. It has been reported that the first loading experience is the most stressful and that the time to load and stress-related behavior frequencies dramatically reduce between the first and the second loading (26). Further studies should be carried out comparing self-loading trained horses with a control group of horses with no experience at all.

Other limitations of this study should be taken into account while interpreting our results. As the interaction with humans is reported to be, *per se*, a stressful event in not accustomed animals [see (27) for a review] the different time of interaction with humans between the two groups should be considered as a limitation of this study. It would have been appropriate to include in our study a sham control group of horses exposed to the same contact time with a person as the trained horses, but without being trained. Due to time and economic constraints, this was not possible and therefore we cannot exclude that being exposed to human contact *per se* might have affected stress-related behaviors during loading procedures. The duration of training (lasting 6 weeks, 3 days per week) was one of the major constraints to the on-farm applicability of the outcomes of the current study, consequently our findings are applicable only to the tested protocol. Future work should envisage shorter yet effective training protocols. Finally, our sample size was relatively small, only one farm was included and there were no repetitions, thus this work should be repeated on a larger number of horses and facilities, and possibly considering additional stress indicators, to ascertain our preliminary results and to compare different loading protocols.

Learning is the relatively permanent change in an animal behavior due to experience (28) and here we hypothesized that, similarly to what was found in the literature on sport horses (11, 16, 17), meat horses trained to load showed different responses to loading procedures compared to untrained control horses. The training protocol applied in this study effectively reduced the time needed to load the horse onto the truck and therefore the entire duration of the loading procedure. This result has practical implication, considering that horses that refuse to load add considerable delays in travel and increase the risk of injuries both for the horses themselves and the personnel (10, 11). Moreover, the variability of the loading duration reflected a higher inter-individual variability in the control group, confirming that trained horses responded to the situation in a more similar manner while untrained ones showed more variable responses. This result could be interpreted in the light of a reduction of the stress caused by loading procedures following training. We found relatively small differences between trained and control horses regarding exhibited behaviors. In addition to the consideration that it was not their first experience, it is worth noting that the usual loading procedures adopted by the farmer, involving minor handling and taking advantage of gregarious behavior of the horses, most likely had a positive effect in keeping the loading-related stress at a minimum in all the considered subjects, minimizing the differences between groups. However, it is also worth to highlight that some behaviors (such as rear, kick, mount, paw, defecate) recognized to be stress related (10) were displayed almost exclusively by horses of the control group. Even a very low frequency of those behaviors should be considered a risk, since they are associated with injuries both to horse and horse handlers (17). Therefore, habituation to load and self-loading training using positive reinforcement should be practiced as early as possible in meat horses, as already suggested in sport horses (11), in order to reduce the stress which the horses are subjected during transport procedures and the related risks. Notwithstanding all the limitations aforementioned, to the authors' knowledge, this is the first study documenting the effects of self-loading training in meat horses. Our results are useful to enhance the welfare of meat horses confirming what reported by a growing number of research focused on the association between training for loading/traveling and transport related behavioral problems in performance horses. Our study provides the basis for future larger-scale studies assessing the impact of loading training on the welfare of horses during the entire transport and at slaughter. It would be relevant to include the assessment of additional animal based indicators such as heart rate, eye thermographic evaluation, cortisol levels.

## Conclusion

Self-loading using positive reinforcement seems to be effective in reducing loading time and the occurrence of behaviors indicative of loading problems in meat horses. Further studies, conducted with a bigger sample size and on several different facilities are needed in order to confirm these results. Our findings provide the basis for future studies looking into streamlining of a feasible protocol to ensure that these principles of loading meat horses are used, which will help improve the welfare of meat horses.

## Ethics Statement

This study was conducted in compliance with the Directive 2010/63/EU Of The European Parliament and of the Council of 22 September 2010 on the protection of animals used for scientific purposes and followed the requirements of the International Society for Applied Ethology (ISAE). Ethical Guidelines; no animals underwent more than a minimal distress. No animals were transported in order to record data for the purposes of this study. Transports were conducted in compliance with Council Regulation (EC) No 1/2005 of 22 December 2004 on the protection of animals during transport and related operations. Verbal informed consent was gained from the farmer prior to taking part in this research. Written consent was deemed unnecessary as no personal details of the participants were recorded.

## Author Contributions

FD, LB, GD, and MM contributed conception and design of the study. FD, CC, AD, and RL performed the experiment. FD and MM performed the statistical analysis. FD wrote the first draft of the manuscript. FD, LB, CC, AD, GD, RL, BP, and MM wrote sections of the manuscript. All authors contributed to manuscript revision, read, and approved the submitted version.

### Conflict of Interest

The authors declare that the research was conducted in the absence of any commercial or financial relationships that could be construed as a potential conflict of interest.
